# TGF-*β* Polymorphisms Are a Risk Factor for Chagas Disease

**DOI:** 10.1155/2018/4579198

**Published:** 2018-02-18

**Authors:** Roberto Rodrigues Ferreira, Fabiana da Silva Madeira, Gabriel Farias Alves, Mayara da Costa Chambela, Eduardo de Oliveira Vaz Curvo, Aline dos Santos Moreira, Renata Almeida de Sá, Leila Mendonça-Lima, Pedro Hernan Cabello, Sabine Bailly, Jean-Jacques Feige, Tania Cremonini Araujo-Jorge, Roberto Magalhães Saraiva, Mariana Caldas Waghabi

**Affiliations:** ^1^Laboratório de Genômica Funcional e Bioinformática-Instituto Oswaldo Cruz, Fundação Oswaldo Cruz (Fiocruz), Rio de Janeiro, RJ, Brazil; ^2^Laboratório de Pesquisa Clínica em doença de Chagas, Instituto Nacional de Infectologia Evandro Chagas, Rio de Janeiro, RJ, Brazil; ^3^Laboratório de Genética Humana-Instituto Oswaldo Cruz, Fundação Oswaldo Cruz (Fiocruz), Rio de Janeiro, RJ, Brazil; ^4^Laboratório de Genética, Universidade do Grande Rio (Unigranrio), Rio de Janeiro, RJ, Brazil; ^5^INSERM, U1036, 38000 Grenoble, France; ^6^University Grenoble-Alpes, 38000 Grenoble, France; ^7^CEA, BIG-Biologie du Cancer et de l'Infection, 38000 Grenoble, France; ^8^Laboratório de Inovações em Terapias, Ensino e Bioprodutos-Instituto Oswaldo Cruz, Fundação Oswaldo Cruz (Fiocruz), Rio de Janeiro, RJ, Brazil

## Abstract

Transforming growth factor *β*1 (TGF-*β*1) is an important mediator in Chagas disease. Furthermore, patients with higher TGF-*β*1 serum levels show a worse clinical outcome. Gene polymorphism may account for differences in cytokine production during infectious diseases. We tested whether *TGFB1* polymorphisms could be associated with Chagas disease susceptibility and severity in a Brazilian population. We investigated five single-nucleotide polymorphisms (−800 G>A, −509 C>T, +10 T>C, +25 G>C, and +263 C>T). 152 patients with Chagas disease (53 with the indeterminate form and 99 with the cardiac form) and 48 noninfected subjects were included. Genotypes CT and TT at position −509 of the *TGFB1* gene were more frequent in Chagas disease patients than in noninfected subjects. Genotypes TC and CC at codon +10 of the *TGFB1* gene were also more frequent in Chagas disease patients than in noninfected subjects. We found no significant differences in the distribution of the studied *TGFB1* polymorphisms between patients with the indeterminate or cardiac form of Chagas disease. Therefore, −509 C>T and +10 T>C *TGFB1* polymorphisms are associated with Chagas disease susceptibility in a Brazilian population.

## 1. Introduction

Chagas disease, caused by the protozoan parasite *Trypanosoma cruzi* [1], originally confined to the American continent, is increasingly becoming a global health problem [2]. WHO estimates that 6-7 million people are infected with *T. cruzi*, mostly in Latin America, and more than 25 million live in risk areas [3]. The natural history of Chagas disease includes two phases: a short acute phase followed by a chronic phase [4], which can be classified into cardiac, digestive, or indeterminate forms [5]. Up to 20–30% of patients with chronic Chagas disease present or will progress to the cardiac form that evolves with high mortality [6]. The Chagas disease cardiac form is characterized by an inflammatory response and progressive heart tissue damage which trigger cardiac remodeling and myocardial fibrosis [7]. Several molecules, such as soluble cytokines and growth factors, may regulate myocardial fibrosis by a complex set of interactions, and the profibrotic protein transforming growth factor-beta (TGF-*β*) is one of those major mediators since it induces fibroblasts and other cell types to synthesize the extracellular matrix (ECM) [8].

TGF-*β* is a homodimeric protein member of a superfamily of polypeptide growth and differentiation factors. In addition to its important function in the fibrotic process, TGF-*β* is also a multifunctional cytokine that works as a physiological on-off switch and triggers a great variety of biological functions with strong effects on the immune response, cell proliferation, differentiation, and cell death [9]. We described important observations about the role of TGF-*β* during *T. cruzi* infection: (i) patients with the Chagas disease cardiac form present TGF-*β* serum levels higher than do patients with the indeterminate form [10], (ii) patients with higher TGF-*β* levels present a worse clinical outcome [11], (iii) *T. cruzi*-infected mice during the acute phase overexpress TGF-*β* receptors and present an elevated activity of its downstream pathway [12], (iv) pharmacological inhibition of the TGF-*β* type I receptor greatly reduces cardiomyocyte invasion by *T. cruzi* in vitro [13], and (v) treatment of *T. cruzi*-infected mice by a single dose of a TGF-*β* receptor inhibitor decreases parasitemia and mortality and prevents heart damage [14, 15]. These data corroborate the importance of TGF-*β* in the development and maintenance of cardiac damage in response to *T. cruzi* infection.

The *TGFB1* gene is located on human chromosome 19 long arm (subbands q13.1) [16]. Several single-nucleotide polymorphisms (SNP) were described in the promoter and coding regions of the *TGFB1* gene [17–19]. Literature reports have already associated an elevated serum concentration of TGF-*β*1 with *TGFB1* SNP: *TGFB1* promoter −509 C>T polymorphism (rs1800469) was linked to a higher TGF-*β* circulating level [20, 21]. Therefore, individual predisposition to produce elevated TGF-*β* due to polymorphisms could be correlated with increased disease risk, such as fibrotic lung disease [22]. The frequencies of the “T” allele in −509 C/T, the “C” allele in 868 T/C (codon +10, Leu to Pro), the “C” allele in 913 G/C (codon +25, Arg to Pro), and the “T” allele in 11929 C/T (codon +263, Thr to Ile) *TGFB1* polymorphisms were higher in patients with acute myocardial infarction [23]. On the other hand, no marked difference with the *TGFB1* polymorphism in codon 10 was observed in left ventricle hypertrophy in a Chinese hypertensive population while there was a significant difference at codon +25 [24]. Very interestingly, five SNP in the *TGFB1* gene of known or suggested functional significance (−988 C>A, −800 G>A, −509 C>T, +10 T>C, and +263 C>T) were studied in a Peruvian and a Colombian population seropositive for Chagas disease versus seronegative. A significant difference was found in the distribution of the *TGFB1* +10T and +10C alleles between patients and noninfected controls, suggesting that the *TGFB1* polymorphism at codon +10 may be involved in a differential susceptibility to *T. cruzi* infection in patients from Peru and Colombia [25]. In the present study, we aimed to check if this association could also be observed in the Brazilian population and to explore if differences in *TGFB1* polymorphism distribution could be observed between patients with the indeterminate and cardiac forms of Chagas disease.

## 2. Material and Methods

### 2.1. Patients

Patients with chronic Chagas disease with the indeterminate or cardiac form followed at the outpatient service of the Evandro Chagas National Institute of Infectious Diseases were included in this cross-sectional study. Noninfected subjects were individuals with a positive epidemiological history for Chagas disease, born and/or living in endemic areas but negative for Chagas disease serology with the same age range of patients. Chagas disease was diagnosed by a positive result in two different serological tests using previously published criteria [26].

All participants gave written informed consent before their entry in the study, which was approved by the local ethical committee under number 02826212.6.0000.5262 and conforms to standards currently applied by the Brazilian National Committee for Research Ethics.

Subjects with any of the following conditions were excluded from the study: previous treatment with benznidazole, coinfectious diseases, pregnancy, autoimmune diseases, cancer, and associated cardiovascular diseases that hampered the classification of the cardiac form of Chagas disease or the associated digestive form of Chagas disease.

During the study, all participants were clinically evaluated and underwent electrocardiogram and echocardiogram. Chagas disease patients were classified according to the current Brazilian consensus into the following: indeterminate form (no evidence of cardiac involvement) or cardiac form stage A (asymptomatic with isolated changes in the electrocardiogram), stage B (asymptomatic with segmental or global left ventricular systolic dysfunction on the echocardiogram), stage C (symptomatic heart failure), or stage D (end-stage heart failure) [27]. They were divided into four groups: (1) noninfected subjects, (2) patients with the indeterminate form, (3) patients at stage A or B, and (4) patients at stage C or D of the cardiac form. Patients with Chagas disease followed at our outpatient facility received standard treatment following the guidelines published by the Brazilian Cardiology Society [28].

### 2.2. TGFB1 SNP

Genomic DNA from all participants was purified by standard methodology (DNeasy® Blood & Tissue Kit, Qiagen, USA). We analyzed five SNP in the *TGFB1* gene: −800 G>A and −509 C>T in the promoter region, codon +10 T>C and codon +25 G>C in exon 1, and codon +263 C>T in exon 5. All polymorphisms were analyzed based on polymerase chain reaction followed by sequencing with a specific group of primers. Primer sequences shown in Table 1 were designed based on RefSeq (NG_013364) and obtained from Invitrogen™. Amplified products were purified using the illustra™ GFX™ PCR DNA and Gel Band Purification Kit (GE Healthcare, USA).

### 2.3. DNA Sequencing of the Specific SNP

Sequencing of the specific SNP was used to detect the polymorphism at promoter positions −800 and −509 and codons +10, +25, and +263 of the human *TGFB1* gene. The DNA sequencing reaction was performed using the BigDye® Terminator v3.1 Cycle Sequencing Kit (Applied Biosystems), following the manufacturer's protocol. Data were generated with an automated instrument ABI PRISM® 3730*xl* Genetic Analyzer, Applied Biosystems. The sequence data were assembled and edited electronically with the BioEdit program v7.2.5 and were compared with the human TGF-*β*1 reference sequence (NG_013364).

### 2.4. Statistical Analysis

Calculations were done using the statistical software SPSS Statistics, Version 22. The sample size to test the difference in *TGFB1* polymorphism prevalence between patients with the indeterminate and cardiac forms of Chagas disease was calculated using the known 70% prevalence of the C allele of the +10 T>C polymorphism and 40% of the T allele of the −509 C>T polymorphism in a Latin American population [25]. Other polymorphisms were rare [25]. Calculating for a 35% increase in the prevalence of the C allele of the +10 T>C polymorphism and of the T allele of the −509 C>T polymorphism in patients with the cardiac form and a correspondent 35% decrease in the prevalence of these alleles in patients with the indeterminate form, with 5% significance and 80% power and a 1 : 1 ratio, we would need 13 patients in each group to test the difference in +10 T>C polymorphism prevalence and 47 patients in each group to test the difference in −509 C>T polymorphism prevalence. We decided to use the minimum sample size that would be needed to test both −509 C>T and +10 T>C polymorphisms. Therefore, we designed the study to include 50 patients in the indeterminate group, 50 patients in stages A + B of the cardiac form, and 50 patients in stages C + D of the cardiac form.

The sample size calculation to study Chagas disease susceptibility was done taking into consideration the described association between the +10 T>C polymorphism and Chagas disease susceptibility in a Latin American population [25]. Considering the known prevalence of 41% [29] of the C allele of the +10 T>C polymorphism in normal subjects from Brazil and of 70% of the C allele of the +10 T>C polymorphism in a Latin America Chagas disease population [25], with 5% significance and 80% power and 1 : 2 ratio, we would need 34 subjects without Chagas disease and 67 patients with Chagas disease. The Hardy-Weinberg equilibrium was evaluated through the *χ*^2^ test. Populations were in Hardy-Weinberg equilibrium, except for +10 T/C SNP in noninfected (*P* = 0.0453) and Chagas disease (*P* = 0.0006) groups and for −509 C/T SNP in the noninfected group (*P* = 0.0213).

Continuous variables were expressed as mean ± standard deviation (SD) and discrete variables as percentages. All continuous variables passed the standard tests of normality (Kolmogorov-Smirnov test) allowing the use of parametric tests. Data between groups were compared using ANOVA followed by Student-Newman-Keuls post hoc analysis. A comparison of allele frequencies between noninfected subjects and patients with Chagas disease was done using 2 × 2 contingency tables and the chi-square and Fisher's exact tests, when appropriate. A comparison of allele frequencies between patients with the indeterminate and cardiac forms was done using 2 × 2 contingency tables and the chi-square test. Odds ratios (OR) were calculated by Woolf's method with a 95% confidence interval, with Haldane correction [30, 31]. Adjustment for multiple comparisons was performed following the method proposed by Benjamini and Hochberg. The null hypothesis was rejected at *P* < 0.05.

## 3. Results

### 3.1. Participants

A total of 219 patients consented to participate in the study. Blood samples from fourteen patients were not collected. Five patients were excluded due to previous treatment with benznidazole (1), hepatitis C virus coinfection (1), hemolysis of the blood sample (1), and associated digestive form of Chagas disease (2). The final studied population consisted of 200 subjects: 48 noninfected counterparts, 53 patients with the Chagas disease indeterminate form, 49 patients at stage A or B, and 50 patients at stage C or D of the cardiac form.

Most participants were born in the Brazilian northeastern region (69%), followed by those born in the southeastern region (26%) and the north, south, and Midwest regions (4%, combined). Patients with the cardiac form were older than patients with the indeterminate form and noninfected subjects. No significant differences were observed in gender, hypertension, diabetes mellitus, coronary artery disease, dyslipidemia, and smoking habit distribution among the studied groups (Table 2). The left ventricular ejection fraction was lower among patients with the cardiac form than in patients with the indeterminate form. Patients at stage C or D presented a left ventricular ejection fraction lower than all other groups did, and most patients of this group had severe left ventricular systolic dysfunction (Table 2).

### 3.2. Association between TGFB1 SNP and Chagas Disease Susceptibility

As the Brazilian population was formed by an admixture of three different ancestries, Amerindian, European, and African [32], we investigated if the frequencies and distribution of mutant and wild-type alleles for the five analyzed SNP would differ between the population from northeast and southeast regions. We found no significant difference in the distribution of any of the five studied *TGFB1* SNP among individuals from these two regions (−800 G/A, *P* = 0.534; −509 C/T, *P* = 0.297; +10 T/C, *P* = 0.713; +25 G/C, *P* = 0.163; and +263 C/T, *P* = 0.514). Thus, we analyzed the association between *TGFB1* SNP and Chagas disease independently of the region the patient was born.

**Figure 1:**
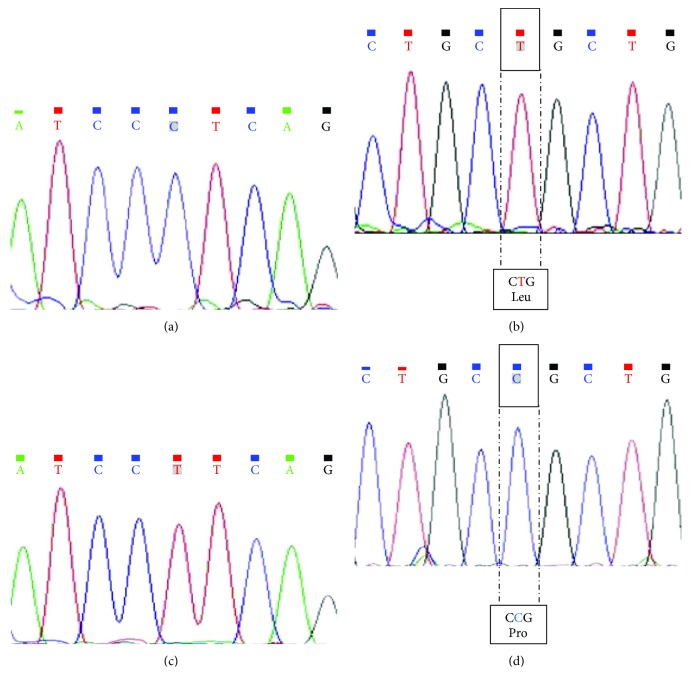
Electropherogram analysis showing the sequencing results for two *TGFB1* regions: −509 C/T and codon +10 T/C. Examples of electropherograms without polymorphism in the −509 C/T region (a) and in codon +10 T/C (b) and with polymorphism in the −509 C/T region (c) and in codon +10 T/C (d). In (b) and (d), amino acid changes are indicated inside the boxes.

We found a significant difference in the distribution of the *TGFB1* −509 C/T and +10 T/C variants between noninfected subjects and patients with Chagas disease. Examples of electropherograms of −509 C/T and +10 T/C sequencing with or without genetic polymorphisms are shown in Figure 1. The genotypes CT and TT at position −509 of the *TGFB1* gene were more frequent in patients with Chagas disease than in noninfected subjects (*P* = 0.0003). Patients heterozygous or homozygous for this allele had an increased risk of Chagas disease (Table 3). Similar findings were observed for the genotype T/C at codon +10 of the *TGFB1* gene: patients heterozygous or homozygous for this allele also had an increased risk of Chagas disease. Moreover, the frequencies of the “T” allele in −509 C/T and of the “C” allele in +10 T/C *TGFB1* polymorphisms were higher in patients with Chagas disease (Table 3). Thus, −509 C/T and +10 T/C *TGFB1* polymorphisms were associated with Chagas disease susceptibility in this Brazilian population (Table 3). On the other hand, there was no significant difference in the distribution of the other *TGFB1* gene polymorphisms (−800 G>A, +25 G>C, and +263 C>T) between noninfected subjects and patients with Chagas disease (Table 3).

As the genotype distribution of the +10 T/C *TGFB1* polymorphism was in Hardy-Weinberg disequilibrium in the Chagas disease group and was borderline in the noninfected subject group probably due to the small sample size, we performed an analysis including data obtained by Calzada et al. [25]. First, we performed an analysis to verify if the genotype distribution was different between Peruvian and Colombian populations. We observed no significant differences between both populations; thus, we could then unify Peruvian with Colombian populations to compare the +10 T/C genotype distribution with the Brazilian population. After including all samples from Colombian, Peruvian, and Brazilian populations, the genotype distribution of the +10 T/C *TGFB1* polymorphism was in Hardy-Weinberg equilibrium among the different groups evaluated in all cohorts. Then, we evaluated the heterogeneity test and observed no significant differences between all samples. This analysis confirmed that allele C and genotypes TC and CC at codon 10 of the *TGFB1* gene were significantly increased in Chagas disease patients (Table 4). Thus, we observed that the joint analysis shown in Table 4 shows that the relative risk from allele C is 46% higher than that from allele T (*P* = 0.0003) when including all populations.

### 3.3. Association between TGFB1 SNP and Chronic Chagas Disease Forms

There were no significant differences in the distribution of any of the studied *TGFB1* SNP among patients with the indeterminate form, stage A or B, and stage C or D of the cardiac form (Table 5). Therefore, the studied polymorphisms were not associated with the severity of chronic Chagas disease.

## 4. Discussion

Involvement of cytokines in the pathogenesis of Chagas disease has been widely demonstrated, and the association of SNP in cytokine genes with Chagas disease was also described [33]. Among studied cytokines, some were associated with general susceptibility to *T. cruzi* infection (IFN-*γ*, MIF, IL-4, TNF, TGF-*β*, and IL-18) and others with cardiomyopathy development (TNF, IL-1, BAT1, MCP-1, LT-*α*, IL-12, and IL-10) [34]. We studied the involvement of TGF-*β* with Chagas disease since the late '90s, and our contribution was recently summarized [35]. Results in animal models indicated that TGF-*β*1 facilitates parasite cell invasion [36] and intracellular survival and multiplication [37], while it inhibits immune response against parasites [38] and induces myocardial fibrosis [39]. In clinical studies, we and others demonstrated that patients with the cardiac form present higher TGF-*β*1 serum levels than do patients with the indeterminate form [10, 40, 41] and that active TGF-*β*1 is present in the myocardium of patients with advanced stages of the cardiac form [10, 42]. Moreover, TGF-*β*1 presents a prognostic value in patients with Chagas disease [11]. On the other hand, others demonstrated that TGF-*β*1 mRNA expression in the myocardium was similar between patients with Chagas heart disease and controls [43] and that TGF-*β*1 serum levels were similar between patients with heart failure due to Chagas disease and controls [44]. Therefore, clinical studies are still needed to elucidate TGF-*β*1's role in Chagas disease pathogenesis and progression.

A previous work has already associated the *TGFB1* polymorphism at codon 10 to Chagas disease susceptibility in Colombian and Peruvian cohorts [25]. Still, it is important to understand if polymorphisms in the *TGFB1* gene are also implicated in Chagas disease susceptibility in Brazil, as the Brazilian population presents a different genetic background from that of other Latin American countries [45]. Furthermore, we also wanted to study the association of polymorphisms in the *TGFB1* gene with the different clinical forms of Chagas disease and with the severity of the cardiac form [46, 47].

Our results show that subjects carrying the TC or CC genotype in codon +10 present a higher risk of developing Chagas disease. This is in accordance with the results obtained in Colombian and Peruvian cohorts [25]. Moreover, we also found that the CT or TT genotype at position −509 was associated with Chagas disease susceptibility in the Brazilian population, which was not the case in Colombian and Peruvian cohorts [25]. These results further support previous experimental models indicating the importance of the TGF-*β* signaling pathway in the susceptibility to *T. cruzi* infection and disease development [10, 12–15, 36, 48]. A polymorphic pattern of the *TGFB1* gene could contribute to an early increase in TGF-*β* levels after *T. cruzi* infection, favoring parasite entry and replication inside cells and establishment of chronic *T. cruzi* infection. It would be interesting to measure TGF-*β* levels during the acute phase of the human disease to correlate its increase with Chagas disease outcome. However, as previously described in the Colombian and Peruvian cohorts [25], there was no difference in the genotype and allele distribution of −509 C/T and +10 T/C *TGFB1* genetic variants among patients with the indeterminate and cardiac forms, suggesting that the *TGFB1* SNP was not associated with the severity of chronic Chagas disease. In fact, the myocardium from individuals with the Chagas disease cardiac form displays a similar number of TGF-*β*-producing inflammatory cells regardless of the presence or not of heart failure [49]. Therefore, more studies will be needed to clarify TGF-*β*'s role in Chagas disease progression.

One of the strengths of this study is the inclusion of noninfected subjects with a positive epidemiological history for Chagas disease in order to study Chagas disease susceptibility. Another strength is the inclusion of an adequate number of patients for each group of Chagas disease from a closely followed cohort, which allowed rich clinical data and a correct patient classification. However, SNP analysis was not adjusted by other epidemiological/vector-related variables that could affect Chagas disease susceptibility or progression. Moreover, other SNP related to other cytokines that could be related to susceptibility to *T. cruzi* infection (IFN-*γ*, MIF, IL-4, TNF, TGF-*β*, and IL-18) or cardiomyopathy development (TNF, IL-1, BAT1, MCP-1, LT-*α*, IL-12, and IL-10) [34] were not studied.

## 5. Conclusions

This is the first study to demonstrate that the frequencies of the polymorphic CT and TT genotypes at position −509 and the TC and CC genotypes at codon +10 of the *TGFB1* gene were increased in Brazilian Chagas disease patients compared to noninfected subjects. However, the distribution of polymorphisms in the *TGFB1* gene among Chagas disease clinical forms was similar. Thus, we conclude that these *TGFB1* polymorphisms are a risk factor for Chagas disease susceptibility but are not associated with the presentation of the clinical form or the severity of the cardiac form in the chronic phase of the disease [50].

## Figures and Tables

**Table 1:** Primer sequences and expected size of the amplified fragment (bp) used to analyze *TGFB1* gene polymorphisms.

Region	SNP	Base change	dbSNP code	Primer	bp
Promoter	−800	G>A	rs1800468	(F)5′-cagttggcgagaacagttgg-3′	594
	−509	C>T	rs1800469	(R)5′-agaacggaaggagagtcagg-3′	

Exon 01	+10	T>C	rs1800470	(F)5′-attcaagaccacccaccttc-3′	730
	+25	G>C	rs1800471	(R)5′-gctcagtgccatcctcttt-3′	

Exon 05	+263	C>T	rs1800472	(F)5′-tttgctccttccttcctcttc-3′ (R)5′-gttcttacacccagacctcatc-3′	700

**Table 2:** Demographic and clinical characteristics of the study population.

	Noninfected *n* = 48	IND *n* = 53	A + B *n* = 49	C + D *n* = 50
*Age, years*	55 ± 13	52 ± 11	61 ± 10^∗^^†^	58 ± 13^∗^^†^
*Male*	16 (32%)	24 (45%)	16 (33%)	26 (52%)
*Geographic origin*				
North	0	0	0	1 (2%)
Northeast	36 (75%)	42 (79%)	27 (55%)^†^	35 (70%)
Midwest	0	0	1 (2%)	2 (4%)
Southeast	12 (25%)	8 (15%)	21 (43%)^†^	12 (24%)
South	0	3 (5%)	0	0
*Hypertension*	26 (55%)	25 (47%)	26 (53%)	21 (42%)
*Diabetes*	10 (20%)	6 (11%)	4 (8%)	5 (10%)
*CAD*	4 (8%)	1 (2%)	1 (2%)	1 (2%)
*Dyslipidemia*	15 (31%)	12 (23%)	15 (31%)	13 (26%)
*Smoking habits*	2 (4%)	2 (4%)	3 (6%)	1 (2%)
*LVEF, %*	65 ± 10	71 ± 7	63 ± 13^†^	35 ± 11^∗^^†‡^
*Medication*				
ACE inhibitor	—	15 (28%)	20 (41%)	29 (58%)
ARB	—	4 (8%)	17 (35%)	21 (42%)
Spironolactone	—	0	6 (12%)	39 (78%)
Carvedilol	—	0	15 (31%)	48 (96%)
Amiodarone	—	0	7 (14%)	18 (36%)
Furosemide	—	0	8 (16%)	46 (92%)
Digoxin	—	0	2 (4%)	21 (42%)
Warfarin	—	0	9 (18%)	20 (40%)
Hydrochlorothiazide	—	13 (25%)	16 (33%)	11 (22%)
Simvastatin	—	16 (30%)	17 (35%)	20 (40%)

ACE: angiotensin-converting enzyme; ARB: angiotensin receptor blockers; CAD: coronary artery disease; LVEF: left ventricular ejection fraction *n* (%). ^∗^*P* < 0.05 versus noninfected, ^†^*P* < 0.05 versus patients with the indeterminate form, and ^‡^*P* < 0.05 versus patients at stage A or B of the cardiac form.

**Table 3:** Genotype and allele distribution of *TGFB1* polymorphisms among noninfected subjects and patients with Chagas disease.

		Chagas disease *n* = 152 (%)	Noninfected *n* = 48 (%)	OR (95% CI)	*P* value	*P* value after correction for multiple comparisons
	−800 G/A					
Genotype	GG	134 (88.2)	45 (93.75)	Reference		
	AG	17 (11.2)	3 (6.25)	1.69 (0.54–5.26)	0.36	0.63
	AA	1 (0.65)	0 (0.0)	1.01 (0.09–11.46)	0.99	0.99

Allele	G	285 (93.75)	93 (96.88)	Reference		
	A	19 (6.25)	3 (3.13)	1.82 (0.61–5.47)	0.28	0.56

	−509 C/T					
Genotype	CC	10 (6.6)	15 (31.25)	Reference		
	CT	68 (44.7)	16 (33.33)	6.13 (2.41–15.58)	**<0.0001**	**0.00035**
	TT	74 (48.7)	17 (35.42)	6.28 (2.49–15.83)	**<0.0001**	**0.00035**

Allele	C	88 (28.95)	46 (47.92)	Reference		
	T	216 (71.05)	50 (52.08)	2.25 (1.40–3.60)	**0.0001**	**0.00035**

	+10 T/C					
Genotype	TT	14 (9.2)	17 (35.42)	Reference		
	TC	93 (61.2)	17 (35.42)	6.64 (2.75–15.10)	**<0.0001**	**0.00035**
	CC	45 (29.6)	14 (29.17)	3.90 (1.54–9.31)	**0.003**	**0.008**

Allele	T	121 (39.80)	51 (53.13)	Reference		
	C	183 (60.20)	45 (46.88)	1.71 (1.10–2.70)	**0.022**	**0.05**

	+25 G/C					
Genotype	GG	131 (86.2)	43 (89.58)	Reference		
	CG	20 (13.2)	5 (10.42)	1.23 (0.47–3.25)	0.67	0.79
	CC	1 (0. 7)	0 (0.0)	1.01 (0.09–11.38)	0.99	0.99

Allele	G	282 (92.76)	91 (94.79)	Reference		
	C	22 (7.24)	5 (5.21)	1.32 (0.52–3.35)	0.55	0.79

	**+**263 C/T					
Genotype	CC	145 (96.0)	47 (97.92)	Reference		
	CT	6 (3.95)	1 (2.08)	1.41 (0.28–7.04)	0.67	0.79
	TT	0 (0.0)	0 (0.0)	—	—	

Allele	C	296 (98.03)	95 (98.96)	Reference		
	T	6 (1.97)	1 (1.04)	1.40 (0.28–6.83)	0.68	0.79

**(a):** 

Population	Chagas disease		Noninfected		Total	OR	95% CI (OR)	
	C	T	C	T				
Peru	94	42	95	69	300	1.6182	1.0073	2.5994
Colombia	376	174	233	161	944	1.4932	1.1405	1.9526
Brazil	183	121	45	51	400	1.7140	1.0813	2.7025
Total	**653**	**337**	**373**	**281**	**1644**	**1.4598**	**1.1908**	**1.7865**

**(b):** 

Heterogeneity test			
Source	*x* ^2^	df	*P*
Significance	13.3043	1	**0.0003**
Heterogeneity	4.397	2	0.1086
Total	17.7440	3	**0.0005**

**Table 5:** Genotype and allele distribution of *TGFB1* –800 G/A, −509 C/T, +10 T/C, +25 G/C, and +263 C/T polymorphisms among patients with indeterminate and cardiac forms of Chagas disease.

	IND *n* = 53 (%)	A + B *n* = 49 (%)	C + D *n* = 50 (%)	*x* ^2^ (*P*)
	**−**800 G/A			
*Genotype*				
GG	45 (84.91)	46 (86.79)	43 (81.13)	2.15 (0.71)
AG	8 (15.09)	3 (5.66)	6 (11.32)	
AA	0 (0)	0 (0)	1 (1.89)	
*Allele*				
G	98 (92.45)	95 (96.94)	92 (92.00)	2.53 (0.28)
A	8 (7.55)	3 (3.06)	8 (8)	

	−509 C/T			
*Genotype*				
CC	3 (5.66)	4 (7.55)	3 (5.66)	0.58 (0.96)
CT	23 (43.40)	24 (45.28)	21 (39.62)	
TT	27 (50.94)	21 (39.62)	26 (49.06)	
*Allele*				
C	29 (27.36)	32 (32.65)	27 (27.00)	0.97 (0.61)
T	77 (72.64)	66 (67.35)	73 (73.00)	

	+10 T/C			
*Genotype*				
TT	3 (5.66)	8 (15.09)	3 (5.66)	4.08 (0.39)
TC	31 (58.49)	31 (58.49)	31 (58.49)	
CC	19 (35.85)	10 (18.87)	16 (30.19)	
*Allele*				
T	37 (34.91)	47 (47.96)	37 (37.00)	4.11 (0.13)
C	69 (65.09)	51 (52.04)	63 (63.00)	

	**+**25 G/C			
*Genotype*				
GG	47 (88.68)	43 (81.13)	41 (77.36)	1.63 (0.80)
GC	5 (9.43)	6 (11.32)	9 (16.98)	
CC	1 (1.89)	0 (0)	0 (0)	
*Allele*				
G	99 (93.40)	92 (93.88)	91 (91.00)	0.71 (0.70)
C	7 (6.60)	6 (6.12)	9 (9.00)	

	**+**263 C/T			
*Genotype*				
CC	51 (96.23)	47 (88.68)	48 (90.57)	0.01 (0.99)
CT	2 (3.77)	2 (3.77)	2 (3.77)	
TT	0 (0)	0 (0)	0 (0)	
*Allele*				
C	104 (98.11)	96 (97.96)	98 (98.00)	0.01 (0.99)
T	2 (1.89)	2 (2.04)	2 (2.00)	
